# Bronchoalveolar lavage fluid characteristics and outcomes of invasively mechanically ventilated patients with COVID-19 pneumonia in Genoa, Italy

**DOI:** 10.1186/s12879-021-06015-9

**Published:** 2021-04-15

**Authors:** Chiara Dentone, Antonio Vena, Maurizio Loconte, Federica Grillo, Iole Brunetti, Emanuela Barisione, Elisabetta Tedone, Sara Mora, Antonio Di Biagio, Andrea Orsi, Andrea De Maria, Laura Nicolini, Lorenzo Ball, Daniele Roberto Giacobbe, Laura Magnasco, Emanuele Delfino, Luca Mastracci, Rosa Mangerini, Lucia Taramasso, Chiara Sepulcri, Rachele Pincino, Martina Bavastro, Matteo Cerchiaro, Malgorzata Mikulska, Bianca Bruzzone, Giancarlo Icardi, Paolo Frisoni, Angelo Gratarola, Nicolò Patroniti, Paolo Pelosi, Matteo Bassetti

**Affiliations:** 1 Infectious Diseases Unit, San Martino Policlinico Hospital – IRCCS for Oncology and Neurosciences, Genoa, Italy; 2 Anesthesia and Intensive Care, San Martino Policlinico Hospital, IRCCS for Oncology and Neurosciences, Genoa, Italy; 3 Anatomic Pathology Unit, Department of Surgical Sciences and Integrated Diagnostics (DISC), University of Genova, Policlinico San Martino University Hospital, IRCCS for Oncology and Neuroscience, Genoa, Italy; 4 Interventional Pulmonology Unit, Policlinico San Martino University Hospital, IRCCS for Oncology and Neuroscience, Genoa, Italy; 5 Flow Cytometry Unit, Policlinico San Martino University Hospital, IRCCS for Oncology and Neuroscience, Genoa, Italy; 6 Department of Informatics, Bioengineering, Robotics and System Engineering, University of Genoa, Genoa, Italy; 7 Department of Health Sciences (DISSAL), University of Genoa, Genoa, Italy; 8 Hygiene Unit, San Martino Policlinico Hospital – IRCCS for Oncology and Neurosciences, Genoa, Italy; 9 Department of Surgical Sciences and Integrated Diagnostics, University of Genoa, Genoa, Italy; 10 Department of Anesthesia and Resuscitation, Policlinico San Martino Hospital, IRCCS for Oncology and Neuroscience, Genoa, Italy

**Keywords:** COVID-19, Bronchoalveolar lavage fluid, Macrophages, Lymphocytes

## Abstract

**Background:**

The primary objective of the study is to describe the cellular characteristics of bronchoalveolar lavage fluid (BALF) of COVID-19 patients requiring invasive mechanical ventilation; the secondary outcome is to describe BALF findings between survivors vs non-survivors.

**Materials and methods:**

Patients positive for SARS-CoV-2 RT PCR, admitted to ICU between March and April 2020 were enrolled. At ICU admission, BALF were analyzed by flow cytometry. Univariate, multivariate and Spearman correlation analyses were performed.

**Results:**

Sixty-four patients were enrolled, median age of 64 years (IQR 58–69). The majority cells in the BALF were neutrophils (70%, IQR 37.5–90.5) and macrophages (27%, IQR 7–49) while a minority were lymphocytes, 1%, TCD3+ 92% (IQR 82–95). The ICU mortality was 32.8%. Non-survivors had a significantly older age (*p* = 0.033) and peripheral lymphocytes (*p* = 0.012) were lower compared to the survivors. At multivariate analysis the percentage of macrophages in the BALF correlated with poor outcome (OR 1.336, CI95% 1.014–1.759, *p* = 0.039).

**Conclusions:**

In critically ill patients, BALF cellularity is mainly composed of neutrophils and macrophages. The macrophages percentage in the BALF at ICU admittance correlated with higher ICU mortality. The lack of lymphocytes in BALF could partly explain a reduced anti-viral response.

## Background

In December 2019, in China emerged a new coronavirus called severe acute respiratory syndrome coronavirus 2 (SARS-CoV-2) and the new disease caused by this virus is named coronavirus disease 2019 (COVID-19). The spectrum of clinical manifestations of SARS-CoV-2 infection is vast, ranging from asymptomatic or patients with few symptoms to complication of severe viral pneumonia with the acute respiratory distress syndrome (ARDS) [1–4]. An excessive inflammatory response to SARS-CoV-2 is a major cause of disease severity and death and is associated with high levels of circulating cytokines, severe lymphopenia and mononuclear cell lung infiltration [5]. There are two distinct but potentially overlapping pathological subsets [6], the first driven by the virus and the second by the host response. In the pulmonary disease, viral multiplication and inflammation in the lung is prevalent.

In addition, in lungs with characteristic diffuse alveolar damage [1] and in the bronchoalveolar lavage fluid (BALF) [7, 8], monocytes and macrophages were prevalent, with a moderate numbers of multinucleated giant cells, and very few lymphocytes. Most of the infiltrating lymphocytes were CD4-positive T cells [1].

In the peripheral blood, a common feature in many patients with COVID-19, is the presence of a global T cell lymphopenia and this is particularly prominent in patients with more severe disease.

In patients infected by SARS-CoV-2, the lymphopenia of circulating T cells may be linked to their recruitment to inflamed tissues with a consequence of T cell depletion from the secondary lymphoid organs [9]. This finding is consistent with the “primary cytokine” storm induced by viral infection which is mainly produced by alveolar macrophages, epithelial and endothelial cells, rather than those in the “secondary cytokine” storm induced by various subsets of T lymphocytes in late stages of viral infection [10–12].

In the literature, there are few studies that analyze at same time both sites, peripheral blood and BALF cellularity and correlate these values with outcomes and clinical or immunological variables. In some studies that describe data only in one of two sites, the low percentage of patients had ARDS or critical pulmonary infection and the data are reported in few patients invasive mechanically ventilated [13, 14].

The main aim of our study is to describe the BALF cellularity of patients admitted to the intensive care unit (ICU) and requiring invasive mechanical ventilation; the secondary outcome is to describe the BALF findings between survivors vs non survivors patients. As post-hoc analysis, we report the relationships with BALF data, clinical, immunological aspects and peripheral blood values which may predict prognosis.

## Methods

Consecutive, critically ill patients requiring invasive mechanical ventilation for severe COVID-19 pneumonia, aged 18 years or over, admitted between March 5th and April 30th 2020 to the Intensive Care Unit (ICU) at San Martino University Hospital in Genova, Italy, were included in the present study. Confirmed infection was defined as real-time reverse-transcriptase polymerase chain reaction (RT-PCR) positive from a nasal and/or throat swab or BALF according to World Health Organization interim guidance [15] together with signs, symptoms and radiological findings suggestive of COVID-19 pneumonia. The study was carried out in accordance with the principles of the Declaration of Helsinki and approved by the Ethic Committee of Liguria Region (Comitato Etico Regione Liguria) (N. CER Liguria 114/2020 - ID 10420). Informed consent was waived by the Ethic Committee of Liguria Region (Comitato Etico Regione Liguria).

### BALF collection

At admission to ICU, a first BALF was collected in each patient and the following were analyzed: total cellularity (%, 10^3^/ml), all cell subpopulations (lymphocytes, neutrophils, eosinophils, macrophages, monocytes), lymphocyte subtypes (T, B, NK), and T lymphocyte activation as HLA- DR expression.

### Preparation of BALF

Fibroscopy was performed with patients sedated with propofol and midazolam and paralyzed with cisatracurium, otherwise intravenous boluses of midazolam or propofol were administered to provide sedation during flexible bronchoscopy with Ambu® aScope TM 4 Broncho Large 5.8 / 2.8. Chest radiography was performed within 3 h to guide the microbiological examination whereas right middle lobe or lingula were chosen in patients with bilateral pneumonia. We performed BAL by serial 20 ml fractions to a total volume of 100–120 ml of room temperature and 0.9% NaCl. BALF, about 60% of lavage volume, was retrieved by gentle syringe suction and put into sterile containers [16, 17].

### Flow cytometry of Broncho-alveolar lavage

The BALF sample containers were adequately disinfected before being sent to the laboratory. Once received, containers were collected in a ventilated room and the exterior of the containers re- disinfected. All sample handling was carried out by experienced staff who wore protective equipment including protective, disposable aprons, molded protection masks (FFP2), goggles and double layer gloves. Sample volume was noted including appearance, colour and possible contamination with peripheral blood. BALF samples were then filtered through a 70 μm nylon Cell Strainer filters (Thermo Fisher Scientific, Massachusetts, United States) and then centrifuged at 1500 rpm for 7 min. The resulting cell pellet was incubated with monoclonal antibodies (mAbs) in BD TruCount™ tubes for 15 min at room temperature in the dark, followed by the addition of FACS Lysing Solution for 15 min at room temperature. The working panel of mAbs at eight colour assays used for the lymphocyte and monocyte evaluation in BAL samples were the following: CD3 FITC/HLA-DR PE/CD4 PerCP-Cy5.5/CD56 PE-Cy7/CD19 APC/CD8 APCH7/CD15 H450/ CD45 VH500 (BD Biosciences, New Jersey, USA), CD66b FITC/HLA-DR PE/CD3 PerCP-Cy5.5/CD33 PE-Cy7/CD14 APC/CD16 APCH7/CD38 VH450/CD45 VH500 (BD Biosciences, New Jersey, USA). Once washed, samples were acquired within 1 h with a FACSCanto™ II flow cytometer (BD Biosciences, New Jersey, USA). The analysis of cytometric data on BALF samples was performed using BD FACSDiva™ software version 6.1.3 (BD Biosciences, New Jersey, USA). After acquisition and during analysis, the absolute number (cells/μL) of positive cells in the sample can be determined by comparing cellular events to bead events. BD FACSCanto™ clinical software (v2.0 or later) subsequently determines absolute counts.

The gating strategy used in this study is briefly described: debris were excluded on FSC-A and SSC-A plot, then, on FSC-A and FSC-W plot doublets were removed and all leucocyte cells (granulocytes, macrophages-monocytes and lymphocytes) were identified by CD45+ vs SSC-A. The CD4+ and CD8+ T cells were further selected among CD3+ population. Before acquisition, instrument sensitivity was evaluated and monitored over time using the BDTM Cytometer Setting &Tracking system. In order to achieve consistent and comparable data over time, all the BD FACSCanto II cytometers were standardized using BD CS&T beads, creating an Application Setting and defining Target Values (TG). Before any acquisition, PMT voltages were updated using Application Setting and maintenance of TG were verified running beads [18, 19].

### Statistical analysis

For this descriptive observational study no sample size calculations were performed. In the descriptive analysis, categorical variables were summarized by means of numbers and percentages, whereas continuous variables were summarized through median values and interquartile ranges (IQR). Normal distribution variables were compared using the t-test and non-normal distribution continuous variables were compared with the Mann-Whitney test. Categorical variables were compared by the Chi-square test. The Pearson or Spearman correlation analysis was performed to show the correlation between clinical parameters and COVID-19 progression. To verify if there are some immunological aspects which may predict prognosis and distinguish between survivors and non survivors, demographic, clinical variables and laboratory values were tested for their association by means of univariate analysis. To assess the differences between groups, univariate logistic regression analysis with group of treatment as binary dependent variable was adopted. A multivariable regression model was made, with ICU-mortality as dependent variable. Variables were selected to be included in the multivariable model when a *P* < 0.10 was found in the univariable analysis. In addition, the following variables were chosen a priori to be included in the model because of their clinical relevance: age, sex, days from the onset of symptoms to ICU admission, interstitial pattern at chest radiography, PaO2/FiO2 ratio, TCD4+/TCD8+ ratio, neutrophils %, macrophages %. Odds ratios (OR) with 95% confidence intervals (CI) were reported. *P* value ≤0.05 was considered statistically significant. The analyses were performed using SPSS Statistics version 21.0 (IBM Corp., Armonk, NY, USA).

All parameters were collected in a relational database connected through a web-based interface for anonymous and automatic data collection [20]. The pathology laboratory database was used for these data collection.

## Results

The clinical characteristics of 64 enrolled patients are shown in Table 1. Concerning the whole cohort, patients were predominantly male (76.6%), with a median age of 64 years (IQR 58–69); most of them (34, 53.1%) had cardiovascular disease and 6 (9.4%) had chronic obstructive lung disease as comorbidities. The median values of days from symptom onset to ICU admission was 9 (IQR 6–15) and the time in days from viral diagnosis to BALF was 4 days (IQR 2–8). Forty-three out of 64 patients (67.2%) are survivors; at time of data collection, 5 patients are still hospitalized. At ICU admission, the median PaO2/FiO2 ratio in all patients was 155 (IQR 129–241), in survivors 175 (IQR 129–158) and in non-survivors was 157 (IQR 128–216). The mortality in ICU was 32.8%. Concerning the microbiological aspects of BALF, 22 out of 64 patients (34.4%) had positive culture: 14 (63%) *Candida* spp., 3 (14%) *Pseudomonas aeruginosa*, 3 (14%) *Enterobacter aerogenes*, 2 (9%) *Staphylococcus aureus* and 2 (9%) *Klebsiella pneumoniae.* In addition to nasal and/or throat swab, the virological analysis on BALF was performed in 49 patients (74%) and RT-PCR for SARS-CoV-2 resulted positive in all of them.

**Table 1:** Baseline clinical features, laboratory findings, treatment and bronchoalveolar lavage fluid characteristics of overall patients, survivors and non-survivors and univariate analysis

	All patients (***n*** = 64)	Survivors (***n*** = 43) (67.2%)	Non-survivors (***n*** = 21) 21 (32.8%)	***P***
Sex, Male (%)	49 (76.6)	33 (76.7)	16 (76.2)	1
Age, years, median (IQR^a^)	64 (58–69)	61 (55–67)	69 (66–72)	**0.033**
Comorbidities				
Cardiovascular disease	34 (53.1)	23 (53.5)	11 (52.4)	1
Immunodepression^b^ (%)	11 (17.2)	7 (16.3)	4 (19.0)	1
Chronic Kidney disease (%)	4 (6.2)	3 (6.9)	1 (4.7)	1
Cerebrovascular disease (%)	4 (6.2)	2 (4.6)	2 (9.5)	1
Chronic obstructive lung disease (%)	6 (9.4)	2 (4.6)	4 (19.0)	0.084
Days from the onset of symptoms to ICU^c^ admission, median (IQR)	9 (6–15)	10 (7–15)	8 (6–14)	0.699
Days from the SARS-CoV-2 diagnosis to BALF^d^ performing, median (IQR)	4 (2–8)	4 (3–9)	4 (2–7)	0.254
Duration (days) of mechanical ventilation, median (IQR)	10 (7–17)	10 (7–13)	11 (6–18)	0.329
Treatment				
Darunavir/ritonavir (%)	32 (50)	21(48.8)	11 (25.6)	1
Hydroxychloroquine (%)	61 (95.3)	42 (97.7)	19 (90.5)	0.249
Corticosteroids (%)	38 (59.3)	29 (67.4)	9 (42.9)	0.103
Tocilizumab (%)	25 (39)	18 (41.8)	7 (33.3)	0.592
Immunoglobulin (%)	8 (12.5)	8 (18.6)	0	0.178
LWMH^e^ (%)	59 (92.1)	41 (95.3)	18 (85.7)	0.320
Chest radiographic abnormality:				
Interstitial pattern (%)	24 (37.5)	15 (34.8)	9 (42.8)	0.578
Bilateral consolidation (%)	37 (57.8)	25 (58.1)	12 (57.1)	1
Monolateral consolidation (%)	16 (26)	11 (25.6)	5 (23.8)	1
Pleural effusion (%)	3 (4.7)	1 (2.3)	2 (9.5)	0.234
**Peripheral blood values at time of BALF performing**				
IL-6^f^ ng/L, median (IQR)	86 (31–344)	63 (17–175)	172 (60–935)	0.236
IL6/lymphocytes, median (IQR)	296 (73–1704)	114 (52–456)	1269 (261–2599)	0.155
D-dimer μg/L, median (IQR)	1389 (913–2334)	1361 (750–2252)	1704 (1238–2310)	0.937
Ferritin μg/L, median (IQR)	992 (832–1458)	984 (837–1487)	1001 (536–1455)	0.338
LDH^g^μg/L, median (IQR)	309 (274–372)	293 (267–355)	332 (298–396)	0.077
Fibrinogen g/L, median (IQR)	5.7 (3.9–7.3)	5.7 (4.4–7.3)	5 (3.4–7)	0.272
CRP^h^ mg/dl, median (IQR)	86 (22–145)	87 (30–138)	84 (16–157)	0.462
PaO2/FiO2^i^ Ratio	155 (129–241)	175 (129–258)	157 (128–216)	0.935

^a^*IQR* Interquartile Range, ^b^*Immunodepression* hematological and/or solid malignancy, chronic treatment with immunosuppressant drugs, ^c^*ICU* intensive care unit, ^d^*BALF* bronchoalveolar lavage fluid, ^e^*LWMH* low molecular weight heparin, ^f^*IL-6* interleukin-6, ^g^*LDH* lactate dehydrogenase,^h^*CRP* C-reactive-protein, ^i^*PaO2/FiO2* arterial oxygen partial pressure/ fractional inspired oxygen

As description of flow cytometry, cellular BALF characteristics of the whole cohort are reported in Table 2. The median total count of cells (10^3^/ml) was 68 (IQR 20–145), while the main cell type were neutrophils (70%, IQR 37.5–90.5) and macrophages (20%, IQR 7–42). The eosinophils were less than 1% and the monocytes were in median 1% (IQR 0.9–3). Lymphocytes were only 1% of cells and of these 92% (IQR 82–95) were TCD3+. Of the TCD3+ lymphocytes, 52% (IQR 39.5–62.7) were TCD8+, only 20% (IQR 13–32) of total TCD3+ were HLA-DR+. The TCD4+/TCD8+ ratio was 0.6 (0.4–1.2). Conversely, in peripheral blood, the comparison of TCD4+/TCD8+ ratio was not different between survivors and non survivors patients (*p* = 0.430). Median age (*p* = 0.033) was higher, while the median absolute number of peripheral lymphocytes (*p* = 0.012) and absolute number of TCD8+ in peripheral blood (*p* < 0.01) were lower in the non- survivors compared to survivors (Tables 1 and 2).

**Table 2:** Cellularity of bronchoalveolar lavage fluid (BALF) and peripheral blood in all patients, survivors, non-survivors and univariate analysis

	All patients (***n*** = 64)	Survivors (***n*** = 43) (67.2%)	Non-survivors (***n*** = 21) 21 (32.8%)	***P***
**Cellularity of BALF**				
Lymphocytes %, median (IQR^b^)	1 (0.4–3.75)	1 (0.3–2.25)	1 (1–4)	0.502
Neutrophils %, median (IQR)	70 (37.5–90.5)	71 (46–90.5)	63 (36–83.5)	0.408
Macrophages %, median (IQR)	20 (7–42)	20 (7–42)	35 (16.5–56.7)	0.194
TCD3+ %, median (IQR)	92(82–95)	92 (83–94)	89 (79–96)	0.873
TCD4+ %, median (IQR)	30 (22–46.5)	31 (22–47)	29 (23–39)	0.976
TCD8+ %, median (IQR)	52 (39.5–62.7)	53 (37–60)	46 (42–64)	0.995
TCD4+/TCD8+ Ratio	0.6 (0.4–1.2)	0.6 (0.4–1.2)	0.5 (0.3–0.8)	0.495
B CD19+ %, median (IQR)	2 (1–5)	2.5 (1–5)	1 (1–5)	0.205
Natural Killer (CD56 + CD16+) %, median (IQR)	3 (2–10)	5 (2–10)	3 (2.7–13.7)	0.618
**Peripheral blood values at time of BALF**^**a**^ **performing**				
Lymphocytes/mmc, median (IQR)	630 (475–1000)	900 (500–1220)	520 (340–622)	**0.012**
TCD4+/mmc, median (IQR)	317 (190–534)	358 (236–651)	227 (152–430)	0.100
TCD8+/mmc, median (IQR)	82 (39–235)	160 (63–267)	44 (29–87)	**0.007**
R TCD4+/TCD8+, median (IQR)	4.1 (2–5.5)	3.2 (1.4–5)	5 (3.6–6.4)	0.430

^a^*BALF* bronchoalveolar lavage fluid, ^b^*IQR* interquartile range

**Fig. 1:**
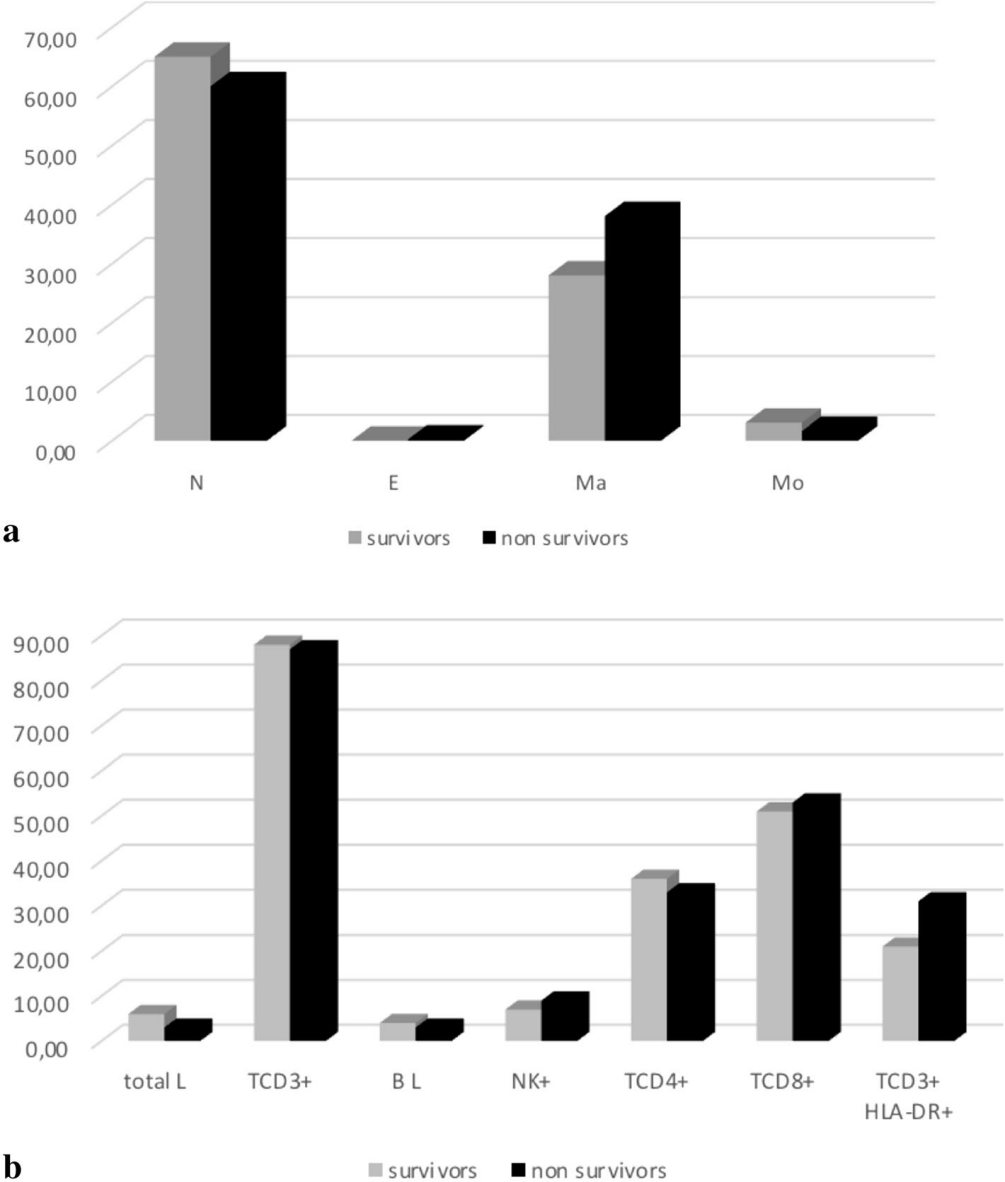
The comparison of percentage of different types of cells in bronchoalveolar lavage fluid in survivors and non-survivors. **a** (neutrophils, eosinophils, macrophages, monocytes). The survivors are represented in grey and non-survivors in black. All values are expressed as percentage. N: neutrophils, E: eosinophils, Ma: macrophages, Mo: monocytes. **b** (total lymphocytes, T CD3+, B, natural killer, T CD4+, TCD8+ and TCD3 + HLA-DR+). The survivors are represented in grey and non-survivors in black. All values are expressed as percentage. L: lymphocytes, TCD3+: lymphocytes T CD3+, B L: B lymphocytes, NK+: natural killer cells, TCD4+: lymphocytes TCD4+, TCD8+: lymphocytes TCD8+, TCD3 + HLA-DR+: activated lymphocytes. In the bronchoalveolar lavage fluid of non-survivors, the median value of macrophage percentages was higher (35%) than in survivors (20%); the TCD4+/TCD8+ ratio was lower (0.5 vs 0.6), and activated lymphocytes (TCD3 + HLA-DR+) were higher in the non-survivors (23% vs 20%) compared to survivors. All the differences are not statistically significant

In the BALF, the median value of macrophage percentages and activated lymphocytes (TCD3 + HLA-DR+) were higher in non-survivors compared to survivors (35% vs 20, and 23% vs 20%, respectively) while the TCD4+/TCD8+ ratio was lower in non-survivors compared to survivors (0.5 vs 0.6, respectively) (Fig. 1Panel A, B). All the differences are not statistically significant.

The mean count of total cellularity in BALF was higher, but not significantly different, between survivors and non- survivors (250 vs 117, 10^3^/ml cells, respectively).

At multivariate analysis only the percentage of macrophages in BALF correlated with ICU mortality (32.8%, *p* = 0.039, OR 1.336, CI95% 1.014–1.759). Post-hoc analysis showed a correlation between macrophages and NK cells in BALF, while the p is not significant (*r* = 0.270, *p* = 0.046), a negative correlation between monocytes and lymphocytes TCD3+ in BALF (*r* = − 0.41, *p* = 0.016) and a negative correlation between %TCD4+ and NK cells in BALF (*r* = − 0.289, *p* = 0.030). The percentage of TCD3+ inversely correlated with blood lactate dehydrogenase (LDH) (*r* = − 0.288, *p* = 0.033). The duration of mechanical ventilation was correlated with percentage of TCD8+ in BALF (*r* = − 0.410, *p* = 0.008), TCD4+/CD8+ ratio (*r* = 0.425, *p* = 0.006) and total lymphocytes TCD3+ (*r* = 0.359, *p* = 0.013) in BALF, respectively. Moreover, the IL-6 values were significantly correlated with the days from the onset symptoms to ICU admission (*r* = 0.489, *p* < 0.001).

## Discussion

In the present descriptive observational study, we found that BALF cellularity of mechanically ventilated patients with COVID-19 pneumonia was characterized mostly by neutrophils, macrophages and a minority of TCD3+ lymphocytes, with a majority of TCD8+, with low percentage of activation (HLA-DR+). In the BALF, the percentage of total cellularity and activated lymphocytes were higher while the TCD4+/TCD8+ ratio was lower in survivors compared to non- survivors. At post-hoc analysis, the percentage of macrophages in the BALF at ICU admittance correlated with higher ICU mortality.

The neutrophilic cellular pattern is typical of ARDS and diffuse alveolar damage (DAD) and as neutrophils release chemokines and cytokines this may explain the generation of the cytokine storm, which is a leading cause of death in patients with severe acute respiratory syndrome [12, 13]. In this phase, recruitment of monocytes contributes to the rapid decline of alveolar patency and promotes ARDS [15].

As reported in recent literature [7], pulmonary involvement of SARS-CoV-2 starts in the second stage with viral multiplication and inflammation in the lungs with lymphopenia in peripheral blood.

There are two possible reasons for the reduction of T cells in patients with COVID-19, lymphocytes are either directly invaded by the virus or they are indirectly damaged by the induced cytokine storm [1].

In our cohort, BALF were collected at a median of 4 days from diagnosis of SARS-CoV-2, at a median of 9 days from the onset of symptoms and for all patients at the day of ICU admission. Therefore, these patients are probably within the second phase of viral infection and we are probably describing, from 9 to 14 days, the overlapping of the viral response phase and the host response inflammatory phase (6).

The peripheral CD8+ T cells lymphopenia in patients admitted to ICU correlates with COVID-19 severity and mortality [21, 22]; TCD4+/TCD8+ ratio is elevated in the peripheral blood compared to BALF in this cohort of patients.

As reported in Table 3 [7, 8, 12–14, 21, 23, 24] there are few data concerning the description of BALF cellularity and peripheral blood in patients with critical pulmonary infection in COVID 19. In one study the analysis of BALF from COVID-19 patients revealed an increase in CD8 T cell infiltrate with clonal expansion [7]. In another study, post-mortem examination of a patient with ARDS in COVID-19 showed lymphocyte infiltration in the lungs [14]. Another study that analyzed post-mortem biopsies from four COVID-19 patients describes that in three patients in lung biopsy were found mononuclear infiltration [8] and in another one recently published, the inflammatory infiltrate, observed in all 38 cases, was largely composed of macrophages in the alveolar spaces (in 24 cases) and lymphocytes in the *interstitium* (in 31 cases) [24].

**Table 3:** Bronchoalveolar lavage fluid (BALF) and peripheral blood cellularity in patients with severe/critical COVID-19

	Total patients	Patients with ARDS^**a**^/critical pulmonary infection (IMV^**b**^)	Peripheral blood cellularity	BALF cellularity or lung biopsy
*Wu C* et al. *JAMA Int Med 2020* [13]	201	84 (*N* = 6, 2.9%)	>Neutrophils < TCD4+ and TCD8+	No data
*Chen T* et al. *BMJ 2020* [12]	274	196 (*N* = 17, 6.2%)	< Lymphocytes >Neutrophils	No data
*Chen G* et al. *J Clin Invest 2020* [21]	21	11 (*N* = 0)	< Lymphocytes >Neutrophils	No data
*Tang X* et al. *Chest 2020* [23]	73	36 (*N* = 14, 19.2%)	< Lymphocytes (TCD4+ and TCD8+)	No data
*Liao M* et al. *Nature Medicine 2020* [7]	9	6 (n.a.^c^)	No data	>Macrophages >Neutrophils < T and NK Lymphocytes
*Xu Z* et al. *Lancet Resp Med 2020* [14]	1	1 (*N* = 1, 100%)	< TCD4+ and TCD8+ Activated Lymphocytes (HLA-DR+)	>Lymphocytes
*Tian S* et al. *Mod Pathol 2020* [8]	4	4 (n.a.)	< Lymphocytes	>Mononuclear cells
*Carsana L* et al. *Lancet Infect Dis 2020* [24]	38	38 (n.a.)	No data	>Macrophages >Lymphocytes

^a^*ARDS* Acute Respiratory Distress Syndrome, ^b^*IMV* Invasive mechanical ventilation, ^c^*n.a.* not available

The peculiarity of our study is the analysis of cellularity both in BALF and in the peripheral blood of mechanically ventilated COVID-19 patients; we describe a reduction of TCD3+ and TCD8+ lymphocytes in BALF with a consequent decrease of TCD4/TCD8 ratio; we found that the majority of cells were neutrophils and macrophages. However, in peripheral blood, we observed an increase of the TCD4+/TCD8+ ratio.

Moreover, cellularity distinguished patients in survivors and non survivors; indeed, we found a reduction of peripheral lymphocytes and absolute TCD8+ in the non-survivor group of patients.

As post-hoc analysis, we found that older age and peripheral lymphopenia, specially TCD8+, correlated with poor outcome. In addition, we found a positively correlation between the duration of symptoms to ICU admission and the peripheral IL-6 values, to strengthen the hypothesis that in this second phase of infection the role of blood immunological markers and BALF cellularity are the main authors.

Fatal cases show persistent and more severe lymphopenia compared with recovered patients, suggesting that a cellular immune deficiency state may be associated with poor prognosis [10]. T cells play a crucial role in viral infections: the TCD4+ cells provide B cell-help for antibody production while TCD8+ cells kill infected cells to reduce viral burden [25, 26]. In our descriptive analysis, we found a reduction of overall lymphocytes, both TCD4+ and TCD8+ with a higher ratio, in the peripheral blood compared to BALF. A negative correlation between monocytes and lymphocytes TCD3+ and also between %TCD4+ and %NK cells in BALF confirm the predominant role of innate immunity cells in this phase of lung damage.

In our study, the percentage of TCD3+ in BALF was inversely correlated with LDH in the blood; this result could explain this phase of lung damage, expressed with increased value of lactate dehydrogenase and the reduction of all lymphocytes in the lung. LDH is an important parameter to measure lung damage and/or lung dysfunction. Indeed, in the CALL score one of the four considered parameters is LDH and this appears to be a simple and accurate model for the prediction of COVID-19 progression to severe cases [27, 28]. This hypothesis is supported by the fact that the duration of mechanical ventilation is inversely correlated with percentage of TCD8+ but shows positive correlation with BALF TCD4+/TCD8+ ratio. Overall the present data suggest that in the second phase COVID-19 with lung viral infection in the lungs there is a relative lack of lymphocytes, in BALF that reflects lymphocyte depletion in the lung [7], with decreased immune responses to control virus replication.

This study has some limitations to be addressed. First, the design of the study is observational and descriptive; second the number of patients is relatively small; third, we did not evaluate CT scan images, but only the radiographic findings and their correlation to BALF data and fourth, we did not evaluate an evolution time for BALF.

## Conclusion

In critically ill patients with COVID-19 pneumonia requiring invasive mechanical ventilation, BALF cellularity is mainly composed of neutrophils and macrophages, with a minority of TCD3+ lymphocytes. The percentage of macrophages in the BALF at ICU admittance correlated with higher ICU mortality. The lack of lymphocytes in BALF, in the second phase of viral infection, could partly explain a reduced anti-viral response.

Further investigation is therefore advisable to gain a better understanding of BALF information to guide efforts aimed at reducing the fatality rate and at clarifying the future evolution of pulmonary follow up of COVID-19 survivors.

## Data Availability

The datasets used and/or analysed during the current study available from the corresponding author on reasonable request.

## Abbrevations

SARS-CoV-2: Severe acute respiratory syndrome coronavirus 2
COVID-19: Coronavirus disease 2019
ARDS: Acute respiratory distress syndrome
BALF: Bronchoalveolar lavage fluid
ICU: Intensive care unit
RT-PCR: Real-time reverse-transcriptase polymerase chain reaction
mAbs: Monoclonal antibodies
IQR: Interquartile ranges
OR: Odds ratios
CI: Confidence intervals (CI)
LWMH: Low molecular weight heparin
IL-6: Interleukin-6
LDH: Lactate dehydrogenase
PCR: C-reactive-protein
PaO2/FiO2: Arterial oxygen partial pressure/ fractional inspired oxygen
IMV: Invasive mechanical ventilation

## References

[1] Zhang W, Zhang Y, Zhang F (2020). The use of anti-inflammatory drugs in the treatment of people with severe coronavirus disease 2019 (COVID-19): the perspectives of clinical immunologists from China. Clin Immunol.

[2] Merad M, Martin JC (2020). Pathological inflammation in patients with COVID-19: a key role for monocytes and macrophages. Nat Rev Immunol.

[3] Rello J, Storti E, Belliato M, Serrano R (2020). Clinical phenotypes of SARS- CoV-2: implications for clinicians and researchers. Eur Respir J.

[4] Channappanavar R, Perlman S (2017). Pathogenic human coronavirus infections: causes and consequences of cytokine storm and immunopathology. Semin Immunopathol.

[5] Mehta P, Mc Auley DF, Brown M (2020). COVID-19: consider cytokine storm syndromes and immunosuppression. Lancet.

[6] Siddiqi HK, Mehra MDR. COVID-19 illness in native and immunosuppressed states: a clinical-therapeutic staging proposal. J Heart Lung Transplant. 2020. 10.1016/j.healun.2020.03.012. 10.1016/j.healun.2020.03.012 PMC7118652 32362390

[7] Liao M, Liu Y, Yuan J, et al. The landscape of lung bronchoalveolar immune cells in COVID-19 revealed by single-cell RNA sequencing. Nat Med. 2020. 10.1101/2020.02.23.20026690.

[8] Tian S, Xiong Y, Liu H (2020). Pathological study of the 2019 novel coronavirus disease (COVID-19) through postmortem core biopsies. Mod Pathol.

[9] Chen Y, Li L (2020). SARS-CoV-2: virus dynamics and host response. Lancet Infect Dis.

[10] Guo X, Thomas P (2017). New fronts emerge in the influenza cytokine storm. Semin Immunopathol.

[11] Shimabukuro-Vornhagen A, Gödel P, Subklewe M, Stemmler HJ, Schlößer HA, Schlaak M, et al. Cytokine release syndrome. J Immunother Cancer. 2018;6(1):56. 10.1186/s40425-018-0343-9. 10.1186/s40425-018-0343-9 PMC6003181 29907163

[12] Chen T, Wu D, Chen H, Yan W, Yang D, Chen G, et al. Clinical characteristics of 113 deceased patients with coronavirus disease 2019: retrospective study. BMJ. 2020;368:m1091. 10.1136/bmj.m1091. 10.1136/bmj.m1091 PMC7190011 32217556

[13] Wu C, Chen X, Cai Y (2020). Risk factors associated with acute respiratory distress syndrome and death in patients with coronavirus disease 2019 pneumonia in Wuhan, China. JAMA Intern Med.

[14] Xu Z, Shi L, Wang Y (2020). Pathological findings of COVID-19 associated with acute respiratory distress syndrome. Lancet Respir Med.

[15] WHO (2020). Laboratory testing for 2019 novel coronavirus (2019-nCoV) in suspected human cases.

[16] Ioanas M, Ferrer R, Angrill J (2001). Microbial investigation in ventilator-associated pneumonia. Eur Respir J.

[17] Vernikos P, Kampolis CF, Konstantopoulos K, Armaganidis A, Karakitsos P (2016). The role of Bronchoscopic findings and Bronchoalveolar lavage fluid cytology in early diagnosis of ventilator-associated pneumonia. Respir Care.

[18] Meyer KC, Raghu G, Baughman RP, Brown KK, Costabel U, du Bois RM, Drent M, Haslam PL, Kim DS, Nagai S, Rottoli P, Saltini C, Selman M, Strange C, Wood B, American Thoracic Society Committee on BAL in Interstitial Lung Disease (2012). An official American Thoracic Society clinical practice guideline: the clinical utility of bronchoalveolar lavage cellular analysis in interstitial lung disease. Am J Respir Crit Care Med.

[19] Klech H, Pohl W (1989). Technical recommendations and guidelines for bronchoalveolar lavage (BAL): report of the European society of pneumology task group. Eur Respir J.

[20] Fraccaro P, Dentone C, Fenoglio D, Giacomini M (2013). Multicentre clinical trials’ data management: a hybrid solution to exploit the strengths of electronic data capture and electronic health records systems. Inform Health Soc Care.

[21] Chen G, Wu D, Guo W, Cao Y, Huang D, Wang H, et al. Clinical and immunological features of severe and moderate coronavirus disease 2019. Clin Invest. 2020;130(5):2620–9. 10.1172/JCI137244. 10.1172/JCI137244 PMC7190990 32217835

[22] Liu J, Li S, Liu J (2020). Longitudinal characteristics of lymphocyte responses and cytokine profiles in the peripheral blood of SARS-CoV-2 infected patient. EBioMedicine.

[23] Tang X, Du R, Wang R (2020). Comparison of hospitalized patients with ARDS caused by COVID-19 and H1N1. Chest.

[24] Carsana L, Sonzogni A, Nasr A, Rossi RS, Pellegrinelli A, Zerbi P, et al. Pulmonary post-mortem findings in a series of COVID- 19 cases from northern Italy: a two-centre descriptive study. Lancet Infect Dis. 2020;20:1135–40. 10.1016/S1473-3099(20)30434-5. 10.1016/S1473-3099(20)30434-5 PMC7279758 32526193

[25] Vabret N, Britton GJ, Gruber C, et al. Immunology of COVID-19: current state of the science. Immunity. 2020. 10.1016/j.immuni.2020.05.002. 10.1016/j.immuni.2020.05.002 PMC7200337 32505227

[26] Diao B, Wang C, Tan Y (2020). Reduction and functional exhaustion of T cells in patients with coronavirus disease 2019 (COVID-19). Front Immunol.

[27] Ji D, Zhang D, Xu J (2020). Prediction for progression risk in patients with COVID-19 pneumonia: the CALL score. Clin Infect Dis.

[28] Grifoni E, Valoriani A, Cei F (2020). The CALL score for predicting outcomes in patients with COVID-19. Clin Infect Dis.

